# STEM Students’ Perceptions of Classical Reading: A Q-Methodology Study on Well-Being-Related Experiences

**DOI:** 10.3390/bs15081074

**Published:** 2025-08-07

**Authors:** Yeonsook Kim, Song Yi Lee, Mikyung Jun, Taeeun Shim

**Affiliations:** 1 Humanitas College, Kyung Hee University, Yongin 17104, Republic of Korea; yeon@khu.ac.kr; 2 Department of Counselling and Coaching, Dongguk University, Seoul 04620, Republic of Korea; songyilee@dongguk.edu; 3 Department of Home Economics Education, College of Education, Dongguk University, Seoul 04620, Republic of Korea; mkjun@dongguk.edu; 4 Department of Education, College of Education, Dongguk University, Seoul 04620, Republic of Korea

**Keywords:** science and engineering students, reading classics, well-being, bibliotherapy, self-growth experience, Q methodology

## Abstract

This study used the Q methodology to examine how Korean science, technology, engineering, and mathematics (STEM) students perceive the experience of reading classical texts and how such experiences relate to their overall well-being. We developed 31 statements for the Q-sorting process and collected data from 39 undergraduate students majoring in science, technology, engineering, and mathematics (STEM). The analysis identified three distinct perception types: type 1—exploratory type, which broadens thinking through diverse perspectives, type 2—experience type, which shares achievement and enjoyment through reading together, and type 3—insight type, which seeks universal values and truth. These findings suggest that, for science and engineering students, reading classics offers a multidimensional experience—encompassing intellectual expansion, relational engagement, and philosophical reflection—beyond conventional academic activities. In particular, the therapeutic dimension of reading, as discussed in bibliotherapy, has emerged as a mechanism that supports self-reflection and emotional resilience. Although each type approached classical reading differently, the participants demonstrated varied perceptions that reflect dimensions of well-being, such as emotional awareness, relational connection, and self-reflection, as expressed through the Q-sorting of pre-defined statements. Based on these results, this study concludes that classical reading can function as a significant mechanism for promoting well-being, offering new directions and practical implications for classical reading education.

## 1. Introduction

The well-being of college students has become a significant social and educational concern in contemporary society. The college years represent a critical developmental stage for exploring identity, gaining independence, and preparing for future life paths (Arnett, 2001). Well-being during this period is not merely an educational issue; it serves as a key psychosocial resource that supports a healthy transition to adulthood. It provides a vital foundation for enhancing individual quality of life, fostering social cohesion, and promoting the psychological well-being of the broader community.

However, the rapid changes in society and the increasingly competitive environment have exacerbated academic stress and adjustment difficulties among college students (H. N. Lee et al., 2019). In Korea, a strong societal emphasis on educational attainment and family expectations regarding social status often intensifies academic stress (Ahn & Baek, 2012; Li et al., 2021). Many students experience psychological burnout during the intense process of preparing for university entrance examinations, and even after entering college, they frequently face a loss of achievement motivation and uncertainty about life direction (Seo, 2011). Furthermore, Korean higher education policy continues to prioritize employment-oriented skill development, while curricula aimed at fostering students’ inner capacities—such as emotional resilience and self-reflection—remain relatively underdeveloped (Y. H. Lee, 2019).

In this context, higher education policies have increasingly emphasized the development of human-centered competencies, including creativity, empathy, ethical judgment, and critical thinking skills (S. M. Lee, 2018). More recently, policy discussions have called for a paradigm shift in higher education from a focus on “job performance” toward “holistic growth” (C. S. Lee, 2025). This policy orientation underscores the continued need for liberal arts education within universities and, in particular, highlights the significance of educational experiences that directly connect to the well-being of individual students.

Recent international discussions, including the Organisation for Economic Co-operation and Development’s (OECD, 2020) “Future of Education and Skills 2030” report, have further emphasized creativity as a core competency for future education. The report highlights that fostering creativity is essential for individual adaptability, societal innovation, and sustainable development. This trend underscores the importance of educational practices, such as classical reading, that foster reflective thinking and imaginative engagement.

Educational contexts and psychological counseling practices have employed various cultural and artistic approaches to alleviate the emotional difficulties and stress experienced by college students. Examples of these interventions include theater therapy (Corcoran, 2018), music therapy (Gwak & Kim, 2012), and bibliotherapy (H. S. Lee, 2019). Among these, bibliotherapy has attracted particular attention as an effective approach for fostering self-understanding and problem-solving skills through engagement with literary texts (Palavan et al., 2022). Bibliotherapy refers to the use of literature for therapeutic and developmental purposes, particularly in fostering self-reflection and emotional healing (H. S. Lee, 2019). In educational settings, it provides an accessible medium through which students can explore personal issues, regulate emotions, and enhance psychological resilience.

Prior research has also shown that college students who engage more actively in reading tend to have higher levels of subjective happiness and life satisfaction (Han & Hwang, 2016). Furthermore, scholars have identified a link between reading and improvements in emotional stability, well-being, and self-efficacy (Ortlieb & Schatz, 2020).

However, much of the existing research on reading has focused on the therapeutic effects of bibliotherapy, primarily through case-based studies or coping strategies for specific problematic situations, often emphasizing short-term outcomes (S. C. Lee, 2013). In contrast, relatively few studies have investigated the emotional transformations that can arise from reading experiences or addressed the broader educational significance of reading as a multidimensional and reflective practice.

Recent studies have highlighted reading as a constructive experience that fosters changes in emotional states and behaviors, extending beyond mere problem-solving (Stanley, 2007). In particular, Brewster (2011) emphasized the need to reinterpret reading as a universal growth experience, arguing that engagement with literary works and self-development books contributes to personal development and social advancement. In line with this perspective, researchers have reconsidered reading as a valuable opportunity for self-reflection and emotional growth, shifting their focus from problem-solving to the pursuit of personal growth and well-being (Currie et al., 2025; Nwagwu & Maxwell, 2025).

This discussion on the positive impact of reading has also expanded to include the reading of classical texts. Scholars recognize classical reading as a meaningful approach to deepening and exploring the educational and psychological benefits of reading. Previous studies have categorized these benefits into three dimensions: learning, aesthetic and emotional engagement, and reflective exploration (Caracciolo & Van Duuren, 2015; Tzoupis, 2024).

The learning dimension encompasses knowledge acquisition and the development of logical and critical thinking skills. The aesthetic and emotional dimensions relate to emotional regulation and artistic sensitivity, while the reflective dimension refers to deep consideration of life’s meaning and the recognition of universal values (Rosenblatt, 1988). Through such multidimensional experiences, classical reading fosters critical thinking and ethical reflection while promoting emotional balance and inner exploration (Oatley, 1995). Moreover, scholars have actively used classical reading as an educational tool that supports psychological recovery and self-growth (B. J. Kim & Jeon, 2016).

Korean higher education has introduced classical reading programs into the regular curriculum since the 2000s to enhance creativity and critical thinking skills (Son, 2013). However, related research often only focuses on the perspectives of educators or educational models and conceptual frameworks, leaving the empirical analysis of learners’ inner changes largely underexplored (Kwon & Yoon, 2021). In practice, classical reading courses in universities often employ evaluation- and task-oriented approaches instead of instructional designs that actively reflect students’ emotional responses and personal growth experiences. Research that seeks to analyze the effects of classical reading based on learners’ actual reading experiences is also rare, with most studies confined to teacher-centered educational models or formal satisfaction surveys. Such approaches fail to fully reflect the educational value of reading and weaken the responsibility of education to support students’ inner development.

The effects of classical reading influence various psychological factors, including learners’ emotional experiences, the pursuit of meaning, and relational interactions. We can systematically interpret these aspects from an academic and universal perspective through the PERMA model (i.e., positive emotions, engagement, relationships, meaning, and accomplishments) proposed by Seligman (2011). Therefore, this study adopts the PERMA model as a theoretical framework to explore how classical reading experiences influence learners’ well-being.

The PERMA model is a multidimensional theory of well-being that provides an integrated framework for explaining psychological well-being and the pursuit of sustainable well-being. Grounded in the principles of positive psychology, fields such as education, counseling, organizational culture, and personal development have widely applied this model (Farmer & Cotter, 2021). In particular, PERMA is well-suited for interpreting complex internal processes, such as classical reading, as it conceptualizes well-being in terms of subjective experiential factors, including emotions, engagement, meaning, and relationships (Kern et al., 2015).

Scholars define each element of the PERMA model as follows. First, “positive emotion” refers to emotional states such as joy, gratitude, and hope, which contribute to psychological resilience (Fredrickson, 2001). Next, “engagement” describes the psychological state of deep focus and absorption when performing challenging tasks, often referred to as flow (Csikszentmihalyi, 1990). “Relationships” encompass positive social connections, emotional bonds, and a sense of belonging that support mental well-being (Baumeister & Leary, 1995). Further, “meaning” involves the pursuit of life purpose and the subjective experience of being connected to something larger than oneself (Ryff, 1989). Finally, “accomplishment” refers to the sense of self-efficacy and satisfaction derived from achieving personal goals and experiencing growth (Bandura, 1997). Collectively, these components serve as a useful analytical framework for interpreting the diverse internal responses elicited by classical reading experiences. This model is particularly appropriate for this study, as it provides a multidimensional framework for understanding the complex emotional, cognitive, and relational responses that classical reading can evoke in students in the science, technology, engineering, and mathematics (STEM) fields.

Classical reading offers a range of experiences, including emotional engagement, interest, immersion, empathetic interaction, reflection on meaning, and a sense of accomplishment. These experiences closely align with the multidimensional elements of well-being. Studies focusing on childhood and adolescence have widely reported on the contribution of reading experiences to emotional well-being. For instance, scholars have shown that shared reading has positive effects on emotional regulation, empathy, and the development of social bonds (Brewster, 2011; Eftimova et al., 2021). Peer reading programs, book clubs, and SEL-based literacy education function as educational tools that promote emotional expression and social skills (L. Yu et al., 2023). These studies demonstrate the strong correlation between reading activities, cognitive improvement, and psychosocial development, thereby offering a basis for interpreting the emotional benefits of classical reading in broader terms.

Specifically, the experience of classical reading closely aligns with the characteristics of literary classical texts, which foster empathy and self-reflective thinking through their narrative structures and allow for open interpretation without a single correct answer. These features enable readers to relate their experiences and emotions to the text and reflect on the meaning of life (J. Park, 2024). Accordingly, reading literary classics can serve as an educational experience that promotes personal growth and the formation of self-identity, moving beyond the simple acquisition of information (McKeon, 2022).

Notably, the experience of classical reading stimulates cognitive flexibility and offers opportunities for science and engineering students (hereafter referred to as STEM students), who are accustomed to standardized problem-solving, to experience shifts in perception. STEM education requires creative thinking and problem-solving skills, but Korean education has often distorted STEM by emphasizing education and standardized learning methods (H. Park et al., 2016). As a result, STEM students have had relatively limited opportunities for reading experiences that foster complex thinking and self-reflection. Therefore, reading literary classics may serve as an educational resource for overcoming these limitations.

In this context, this study selected STEM college students as the target group to examine the impact of classical reading on their well-being. Researchers generally consider STEM students a distinct reading population, as they tend to show low intrinsic motivation for reading and often perceive reading as a task tied to academic performance (H. I. Kim, 2025). Compared to students in the humanities and social sciences, STEM students are typically less emotionally engaged in reading and less likely to read voluntarily. This characteristic suggests that their classical reading experiences may serve as meaningful educational cases for analyzing emotional growth and internal transformation.

Y. H. Lee (2019) demonstrated that bibliotherapy programs for university students positively impacted their self-acceptance, life purpose, and emotional stability, thereby illustrating the practical relevance of exploring emotional outcomes in university readers. However, most existing studies on classical reading focus on general benefits or specific program cases; empirical analyses of emotional and cognitive changes across academic disciplines remain rare. Research targeting the contextual and subjective experiences of STEM students, in particular, is extremely limited.

While few studies have employed Q methodology to examine well-being in STEM students, comparable research using survey-based designs (Freire et al., 2019; Cho & Won, 2024) highlights the role of self-efficacy and reflective experiences in promoting psychological well-being. Building on this gap, the present study employs Q methodology to categorize subjective perceptions and analyze the inner experiences of STEM students as they engage with classical texts. In doing so, it contributes an empirical foundation to the interdisciplinary field of well-being research.

This study examines the impact of classical reading experiences on the well-being and personal growth of STEM college students. From the learners’ perspectives, this study reconsiders these reading experiences as universal growth experiences and explores whether classical reading holds educational significance in promoting emotional well-being beyond extracurricular activities.

In this study, the term “classical reading experiences” refers to reading experiences gained through classical reading courses within the liberal arts curriculum. This definition reflects the educational context in which the participants engage with classical texts.

Accordingly, this study’s research questions are as follows:

1. How do STEM students perceive the classical reading experience?
2. What are the characteristics of the perception types regarding the classical reading experience?

## 2. Methods

This study applied the Q methodology to explore the perceptions of STEM college students regarding the classical reading experience. Q methodology, theorized by Stephenson in 1953, combines quantitative and qualitative approaches by systematically measuring operant subjectivity and analyzing attitudes, beliefs, and values through statistical techniques (Vecchio et al., 2022). Stephenson argued that Q methodology enables a more objective study of subjective behaviors that researchers previously considered impossible or impractical to examine (McKeown & Thomas, 2013). Scholars recognize Q methodology as an effective research method for analyzing small-scale samples and respondent-centered analysis, as it focuses on differences within individuals rather than between individuals in small sample groups (Watts & Stenner, 2012).

Rather than aiming for statistical generalization based on large samples, Q methodology seeks to uncover individuals’ cognitive structures related to specific issues. As such, it focuses on interpretive understanding of intra-group reasoning processes, rather than population-level inference. This approach also redefines traditional concepts of validity and reliability as understood in quantitative research, framing them differently within Q methodology (Ramlo, 2024).

Q methodology considers each participant’s Q-sorting a valid expression of their subjective viewpoint, independent of external criteria. The researcher ensures validity through careful selection of Q-statements based on literature reviews and domain expertise, while the coherent typologies that emerge from the sorting process reflect reliability (Valenta & Wigger, 1997).

This study followed the standard procedures of Q methodology (Table 1).

We extracted a Q concourse by reviewing previous studies and analyzing reflection records related to classical reading. First, we constructed a Q sample to align with this study’s purpose, excluding statements with ambiguous or overlapping meanings. Second, we recruited a P sample, selecting participants appropriate for the research topic. Third, the P sample completed the Q-sorting task by reflecting on their thoughts about the research topic. Fourth, we coded and analyzed the Q-sorting data using the KenQ Analysis Desktop Edition (KADE, version 2.0.1; Ken-Q Analysis, LLC, Charlotte, NC, USA).

Finally, we adhered to ethical standards throughout the research process to ensure reliability and validity and conducted this study after receiving IRB approval (KHGIRB-24-542 (NA)).

### 2.1. Q Concourse

The Q concourse refers to a collection of statements that encompass all possible opinions and viewpoints related to the research topic. It is a process of comprehensively gathering the subjectivity that the researcher aims to examine (H. G. Kim, 2007). In this study, we defined the Q concourse as all statements related to the subjective perceptions of college students regarding classical reading.

To encompass a broad range of perspectives, this study constructed the Q concourse using an extractive method, an approach that collects statements from various academic sources related to classical reading (McKeown & Thomas, 2013). For example, we referenced previous studies on the significant effects of reading on individual growth and social development (Brewster, 2011; H. S. Lee, 2019; Stanley, 2007), the educational importance of reading classics (Son, 2013), and the self-growth of college students (Magolda, 1998, 2004). In addition, we used data generated from the classical reading course—such as test answer sheets, student assignments, presentation materials, and student reflections—to select 175 preliminary statements.

This study employed the PERMA model as a theoretical framework and constructed Q-statements based on the emotional and psychological changes experienced by students after completing a classical reading course. To this end, we analyzed course materials, including test responses, assignments, presentation slides, and reflective essays, to extract shifts in perception. We systematically categorized the extracted content into ten subdomains aligned with the five PERMA elements: emotional arousal, emotional regulation and comfort, immersion and focus, challenge and task engagement, empathy and communication, communal reading experience, existential exploration and self-reflection, philosophical reflection and values, sense of accomplishment, and growth and self-development. This classification reflects how classical reading experiences correlate with various dimensions of well-being at the individual statement level.

Moreover, we did not base the Q-statements on general opinions but on students’ subjective perceptions after the course, thereby functioning as a form of post-experience data. The P-sample participants also took part in the Q-sorting process after completing the course and reflecting on its impact. Thus, although we did not conduct a direct follow-up survey, this study captures the transformed perceptions that followed the reading experience.

For example, the process of developing statements involved analyzing student-generated course materials—such as reflection papers, assignments, and exam responses—and identifying recurring themes and expressions. We frequently observed phrases related to understanding others, deepening empathy, encountering diverse perspectives, and engaging in meaningful dialogue; we synthesized these expressions into core concepts such as “understanding others.” We then generated intermediary phrases and refined them into final statements that reflected the essence of the students’ experiences. We mapped each finalized statement to a relevant subdomain within the PERMA model, ensuring that the content aligned conceptually with the theoretical framework and accurately captured the educational and emotional dimensions of classical reading. We consistently applied this approach across all five PERMA components to maintain coherence between student experiences and the theoretical structure.

### 2.2. Q Sample

The Q sample is the set of statements that participants classify. It plays a critical role in ensuring the reliability and validity of the research findings; the outcomes may vary significantly depending on how the researchers prepare the statements (Parry, 2022). In addition, the process of constructing a Q sample from the Q concourse can vary depending on the theoretical framework of the research question and the degree of researcher intervention (Baek, 2015).

This study constructed the Q sample through the following procedure:

First, we reviewed 175 statements collected from the Q concourse and categorized them into three major areas: the meaning of classical texts, the significance of classical reading lectures, and the effects of classical reading.

Second, we reorganized the categorized statements into the following ten subdomains based on the PERMA model: emotional arousal and emotional regulation (P), immersion and task engagement (E), empathy and communication, and communal reading experience (R), existential exploration and philosophical reflection (M), and accomplishment and self-development (A). This classification structurally reflects how classical reading experiences relate to various dimensions of well-being.

Third, we selected representative and comprehensive statements for each category, resulting in 133 items.

Fourth, we consolidated similar statements, and following a review by two experts experienced in Q methodology, we removed 78 inappropriate items, reducing the final set to 55 statements.

Fifth, four STEM students (two male and two female) reviewed the statements to determine whether they reflected the classical reading experience.

Sixth, we reviewed the subject matter and clarity of each statement, revising them for accuracy and conciseness, which resulted in a set of 31 representative items.

The Q sample construction process involved four researchers with disciplinary expertise in literature education, educational studies, counseling, and human development—fields closely related to higher education and classical reading. Drawing on their teaching experience with STEM students, the researchers initially developed statements through a comprehensive review of prior research and course materials related to classical reading and well-being. We then collaboratively refined each statement through iterative discussions until the data reached theoretical saturation. This process, grounded in domain-specific knowledge and educational practice, contributed to the content and construct validity of the Q sample. Q methodology considers expert review by specialists an important step in enhancing content validity and reliability in the development of the Q sample (Damio, 2016). Accordingly, we also conducted such an expert review in the final stage of statement selection for this study.

### 2.3. P Sample

The P sample refers to the participants who engage in the Q-sorting process and constitutes one of the key elements in the Q methodology. Unlike the R methodology, the P sample in Q methodology does not require strict randomization; instead, it is important to select participants who meet the research purpose (Brown, 1993; Zabala et al., 2018; Silvius et al., 2017). This process enables flexible and purposive sampling based on the principles of adequacy and sufficiency within the framework of Q methodology.

To recruit participants for this study, we posted a recruitment notice on bulletin boards across various STEM-related colleges, including the Colleges of Engineering, Electronics and Information Engineering, Software, Applied Sciences, and Life Sciences. We recruited participants from a pool of students who had previously taken a classical reading course as part of the university’s general education curriculum—39 students volunteered for the study.

We purposively selected the P sample from students who explicitly expressed personal growth through classical reading in their course reflections and assignments. By gender, the sample included 14 females and 25 males. By academic year, the sample consisted of fourteen first-year, seven second-year, fourteen third-year, and fourteen fourth-year students.

The participants in this study were science and engineering students enrolled in two classical reading courses. One course focused on the Korean novel *Land* (*Toji*) by Park Kyung-ni, and the other on the Western tragedy *Oedipus Rex* by Sophocles. Each course focused on a single classical literary work. In the case of *Land*, the instructor assigned selected volumes from the larger multi-volume novel for in-depth reading and discussion. Both courses emphasized in-depth reading, discussion, and reflection. For this study, we analyzed the experiences of students from both classes collectively.

### 2.4. Q-Sorting

This study conducted the Q-sorting process using a distribution framework similar to a normal distribution.

The Q-sorting procedure proceeded as follows. First, we introduced the 31 statements to the participants. Second, the participants read and classified each statement into three categories based on their opinion of each statement: disagree (−), neutral (0), and agree (+). Next, the participants placed the statements with which they disagreed and agreed on the distribution grid according to the degree of agreement, ranging from −4 to +4, placing the statements with which they most strongly agreed or disagreed at the outer edges. They placed neutral statements in the center of the distribution.

Finally, for the statements placed at both extremes (−4 and +4), participants explained, in writing, why they selected those statements. Their explanations contributed to a richer interpretation of each type.

### 2.5. Analysis

Based on the data obtained through the Q-sorting process, we coded the responses by assigning scores ranging from −4 to +4 according to the Q sample distribution grid. We then analyzed the data using principal component analysis and varimax rotation through the KADE V.2.0.1 program. We determined the appropriate number of factors by reviewing the eigenvalues and cumulative variance ratios of each factor and identified the detailed characteristics of each type.

We focused our interpretation of statements by type on those with Z-scores of +1 or higher or −1 or lower, as these values represent strong agreement or disagreement within each type. To deepen our analysis, we also examined statements that demonstrated significant differences between types by reviewing the Q-values, allowing us to identify distinguishing perspectives. Qualitative interview data, which captured participants’ reasoning for their choices, further supported this interpretation (Banasick, 2019; Rahma et al., 2020).

Based on the factor analysis, we reviewed the Z-scores of statements associated with each factor and summarized the defining characteristics of each type. We used the Q-sort values to indicate the degree of agreement and disagreement expressed by participants in each type (Kwok et al., 2024).

## 3. Results

### 3.1. Analysis of Results

The classical reading experiences of Korean STEM students resulted in three types, identified by grouping P samples with similar opinions, thoughts, and attitudes. We analyzed the characteristics of each type and found that these three types explained 50% of the cumulative variance. In general, a cumulative variance of 50% or higher indicates a high level of explanatory power (Watts & Stenner, 2012).

The explanatory power of each type was 35% for type 1, 9% for type 2, and 6% for type 3. The eigenvalues for the three types were 13.52561, 13.52561, and 2.255995, respectively (Table 2).

Table 3 presents the results of the factor analysis for the P sample, including the characteristics of the 39 participants and the factor weights for each type. The analysis of the factor weights of P samples with similar opinions, attitudes, and values regarding the classical reading experience, identified the following as representative participants for each type: P9 (10) for type 1, P38 (6.67231) and P19 (6.3084) for type 2, and P31 (6.12539) for type 3.

### 3.2. Characteristics of Classical Reading Experiences Among STEM Students

This study explored how STEM students perceive their classical reading experiences using Q methodology and identified three distinct perception types as detailed in Appendix A. The interpretation of these types followed the standard approach in Q methodology, which involves analyzing distinguishing statements, prototypical responses, and participants’ qualitative comments. Based on this analysis, we categorized the classical reading perceptions of STEM students into the following three types.

In this process, we highlighted Q-statements with Z-scores equal to or greater than +1.00 or equal to or less than −1.00 as illustrative of the most salient characteristics of each type and presented corresponding Q-sort values. In addition, we included distinguishing statements that were statistically significant (*p* < 0.05; * indicates significance at *p* < 0.01) even if their Z-scores did not exceed ±1.00, as they were meaningful in interpreting the typology.

#### 3.2.1. Type 1: Exploratory Type That Broadens Thinking Through Diverse Perspectives

Our analysis revealed that type 1 participants perceive classical reading as a learning opportunity that broadens their thinking and fosters self-reflection. They actively explore diverse perspectives and highly value deep and critical thinking. Additionally, they engage with complex social and ethical issues and utilize classical texts as a tool for understanding human nature. Rather than focusing on the acquisition of practical knowledge, they tend to view classical reading as a process of self-understanding and intellectual growth. Reflecting these characteristics, we labeled this group the “exploratory type that broadens thinking through diverse perspectives.”

The results of the Q-sorting analysis showed that type 1 strongly agreed with statements such as “Classical reading helps me understand others” (S8, +3), “Classical reading cultivates an attitude of living together” (S9, +3), and “Classical reading rejects the absoluteness of right and wrong” (S3, +2). In contrast, they strongly disagreed with statements such as “Classical reading provides rest and relaxation” (S5, −2) and “Classical reading helps relieve the stress of college life” (S26, −3). Notably, they most strongly disagreed with “Classical reading provides practical knowledge” (S27, −4).

This tendency reflects the characteristics of type 1, who recognize classical reading as a source of enjoyment and relaxation, as well as an intellectual process that broadens thinking and fosters cognitive engagement.

This perception was also evident in the individual responses of the participants. Students classified under type 1 reported that reading classics helped them understand various groups of people and broadened their perspective to consider the viewpoints of others (P24). They also mentioned that they were able to understand the complex situations faced by individuals through the actions and circumstances of the characters (P14). The readings encouraged them to consider multiple perspectives rather than make definitive judgments about right and wrong (P27, P29).

Type 1 did not view classical reading as a means of rest or stress relief. Instead, they reported experiencing cognitive expansion and inner exploration through immersion in classical texts (P9). Some respondents described the classics as a “textbook” for life direction (P5). Others stated that the reading process offered them opportunities to pose questions and engage in contemplation (P10).

In this way, type 1 recognized classical reading primarily as an opportunity for learning and reflection rather than as a source of emotional comfort, demonstrating a tendency to prioritize thinking over emotion. Table 4 summarizes the statements with which type 1 most agreed and disagreed.

#### 3.2.2. Type 2: Experience Type That Shares Achievement and Enjoyment Through Reading Together

We observed that type 2 participants perceive classical reading as a meaningful challenge. They find enjoyment and interest in the process of reading and discussing classics together, and value the sense of achievement and satisfaction that comes from completing these readings. Reflecting these characteristics, we named this group the “experience type that shares achievement and enjoyment through reading together”.

The results of the Q-sorting analysis showed that type 2 strongly agreed with statements such as “Reading classics together is more rewarding than reading alone” (S4, +4), “Classical reading allows me to feel a sense of accomplishment” (S30, +4), and “Classical reading provides rest and relaxation” (S5, +3). In contrast, they strongly disagreed with statements such as “Classical reading makes me reflect on my past” (S13, −3) and “Classical reading breaks down my fixed ideas” (S23, −1).

The participant’s statements also confirmed this tendency. For instance, P38 (7.67) and P19 (6.31), with high factor weights, reported that they encountered new perspectives through reading and discussing classics together, which motivated them to complete lengthy classical texts. Type 2, being familiar with answer-focused learning, recognized that their thinking became more flexible through the free interpretation and discussion of classics (P19).

Participants also described classical reading as a rewarding challenge that brought them a sense of fulfillment. In particular, they noted that reading difficult and lengthy classics together helped them stay engaged and complete the reading, which in turn enhanced their sense of confidence and satisfaction (P21, P17). At the same time, many participants perceived classical reading as an opportunity for relaxation. Unlike their major courses, which focus on formulas and calculations, classical reading offered a different kind of focused attention, providing participants with an opportunity for emotional recharge. (P28, P17).

However, participants explained that they did not often reflect on their past through classical reading (P32) and felt that it was difficult to relate the content of the classics to their lives because the social systems and ways of thinking presented in the texts were different from those of the present (P21).

Type 2 perceived classical reading as a social activity, enjoying the process of reading and engaging in discussion with others. They viewed this experience as a source of personal pleasure and self-reward and reported that it played an important role in sustaining their reading motivation. For these participants, classical reading provided opportunities for social interaction and personal satisfaction, going beyond the framework of academic learning. Table 5 summarizes type 2’s results.

#### 3.2.3. Type 3: Insight Type That Seeks Universal Values and Truth

An analysis of Table 6 reveals that type 3 participants recognize classical reading as a tool for exploring human nature and the truth of life. They believe that reading classics offers opportunities to gain philosophical insights and discover universal values, and they engage with the texts as a way to reflect on ethical issues and establish a sense of direction in life. Reflecting on these characteristics, we labeled this group the “insight type that seeks universal values and truth.”

The results of the Q-sorting analysis showed that type 3 strongly agreed with statements such as “Classical reading broadens my perspective on the world” (S21, +4) and “Classical reading leads to an exploration of human nature” (S2, +4). They also agreed with the statement, “Classical reading encourages multiple perspectives on events or situations” (S14, +2).

The responses of students with high factor weights clearly reflect this perception (P31 [6.13], P26 [5.14]). These participants reported that they were able to explore fundamental themes that are often difficult to encounter in everyday life and to reflect deeply on human nature through classical reading (P31). They recognized that the classics provide insights into human existence across generations and stated that this process led them to consider their life direction and personal values while reflecting on the lives of those who came before them (P26). One participant noted that they were able to examine their value system through the conflicts and choices faced by the characters in the classics (P35), and as a result, they felt they had developed a perspective that connects the past with the present (P36).

On the other hand, type 3 participants disagreed with the statements “Classical reading is especially necessary for STEM students” (S6, −3) and “Classical reading interferes with major studies” (S29, −4). However, their disagreement with the latter was relatively weaker compared to types 1 and 2. Some participants commented that classical reading helped to refresh their overloaded minds from major-related tasks and, instead of interfering, it facilitated cognitive flexibility (P31). Another participant reported that reading classics provided opportunities to cultivate philosophical thinking, which in turn complemented their major research activities (P18).

These responses illustrate the characteristics of type 3, who perceive classical texts as literary works and tools for engaging in essential reflection on human nature and life. Table 6 summarizes the results for type 3.

## 4. Discussion

STEM students commonly reported that classical reading enhanced their well-being, but the patterns of this experience differed by type. The three perception types identified in this study—exploratory type that broadens thinking through diverse perspectives, experience type that gains shared achievement and enjoyment through reading together, and insight type that seeks universal values and truth—each demonstrated distinct characteristics in how classical reading promotes well-being.

This study focused exclusively on classical literary texts and did not include comparisons with contemporary or non-literary genres. The rationale for this focus lies in the distinctive features of classical literature, such as its engagement with timeless themes, philosophical and moral inquiry, and openness to diverse interpretations. These characteristics appeared to foster emotional immersion and deep self-reflection among participating students, which contributed to their experiences of well-being across multiple dimensions. Therefore, we should examine how classical reading affects readers by considering the distinctive characteristics of the texts.

Type 1—the exploratory type that broadens thinking through diverse perspectives—recognized classical reading as an analytical and logical challenge. They engaged with difficult texts to the end and developed confidence in their abilities. This experience led to a psychological shift, fostering self-awareness of their ability to overcome challenges beyond merely completing tasks. The core psychological mechanism at work in this process is self-efficacy, which is the belief in one’s ability to address difficult tasks successfully. It enhances initiative and persistence in task performance, which in turn promotes a sense of accomplishment and engagement (Choi et al., 2014). The learning, reflection, and task engagement reported by type 1 reinforced this self-efficacy, leading to deeper engagement in the learning process and a sustained willingness to take on challenges.

These changes correlate with the elements of engagement and accomplishment in Seligman’s (2011) PERMA model. The engagement they experienced—also referred to as immersion in the context of flow theory (Csikszentmihalyi, 1990; Csikszentmihalyi et al., 2014)—was not merely a state of concentration. It was an intellectual response and an active thinking process when interacting with complex texts. This finding aligns with previous studies, which report that such immersive engagement fosters intrinsic motivation and psychological satisfaction.

This group described their sense of accomplishment as self-trust and satisfaction gained through continuous cognitive challenges rather than result-oriented success, consistent with previous research that links self-efficacy, achievement, and well-being (Cho & Won, 2024; Freire et al., 2019). In this study, the *term* “accomplishment” in type 1 specifically refers to this deep sense of satisfaction driven by cognitive engagement and self-efficacy, *whereas* “achievement” in type 2 reflects emotional gratification and the completion of shared social activities. Despite the shared terminology, the underlying psychological mechanisms and emotional experiences related to well-being differed across the types.

In addition, the proportion of female students in this type was relatively high, and all participants with higher factor weights were female. This finding aligns with previous studies suggesting that certain types of reading experiences may vary by gender (Logan & Johnston, 2010; Jabbar & Warraich, 2023). However, since this study did not specifically analyze gender differences and the sample composition was imbalanced, researchers should approach these interpretations with caution.

Type 2—the experience type that shares achievement and enjoyment through reading together—perceived classical reading as an experience of emotional immersion and relationship building. In particular, they shared emotional responses and strengthened social bonds through collaborative reading and discussion. Empathy for situations and characters in the classical texts extended to recognizing and expressing their emotions, as well as developing relational attitudes that foster understanding and acceptance of the emotions of others. The key psychological mechanism underlying this process is emotional intelligence, which encompasses the ability to recognize and regulate emotions, demonstrate empathy, and engage in interpersonal interactions (Song & Cho, 2018). This process of emotional exploration and relationship building aligns with the positive emotion and relationship components of Seligman’s (2011) PERMA model, as it promotes positive emotional experiences and reinforces social connections among students.

Type 2 participants experienced emotional stability through reading, discussion, sharing, and empathizing with others as facilitated by classical reading. They perceived the class as a place of learning and a space for emotional connection and interaction. This experience aligns with the findings of K. M. Kim and Hong (2016), who suggested that participation in presentations and discussions enhances interest and engagement in class. It is also partially consistent with J. Y. Lee’s (2024) interpretation that the shift from individual reading to shared reading fosters intrinsic motivation. Furthermore, previous studies that emphasized the role of reading activities in psychological stability through emotional regulation and social bonding (Fuller & Sedo, 2013; Álvarez-Álvarez, 2016; Eftimova et al., 2021) support the findings of this study regarding type 2.

In summary, the classical reading experiences of type 2 contributed to the formation of relational bonds and psychological stability through emotional awareness and exchange. These findings suggest that participants perceive classical reading as an educational resource that can support social belonging and psychological well-being.

Type 3—the insight type that seeks universal values and truth—engaged in reflection on ontological questions and life direction through classical reading. By projecting the choices and conflicts of characters onto themselves, participants discovered meaning through the process of interpreting and integrating their experiences. These experiences align conceptually with the meaning element of Seligman’s (2011) PERMA model. In particular, type 3 participants did not simply pursue meaning; they reported the discovery and internalization of meanings personally connected to their lives. This finding contrasts with previous studies (Dogan et al., 2012; Dezutter et al., 2014), which suggested that meaning pursuit does not directly contribute to well-being. Instead, it supports Kleiman and Beaver’s (2013) conclusion that meaning discovery fosters psychological well-being.

This process of constructing meaning supports the view that individuals strengthen their psychological stability and well-being by reconstructing diverse life experiences into a coherent narrative that provides unity and purpose (Arslan et al., 2022). Type 3 participants reinterpreted their values and life contexts through classical characters and narratives, forming an integrated understanding of the self. Additionally, the emotional identification expressed as “this is my story” illustrates the potential of educational reading approaches where readers actively engage with texts and make personal meaning from them (Jeong, 2017). In other words, type 3 participants experienced a narrative reconstruction of life’s meaning through classical reading, which reflects a pathway to psychological well-being centered on the meaning element of the PERMA model.

Each type of classical reading experience identified in this study demonstrates conceptual correspondence with the five elements of the PERMA model, which explains the psychological mechanisms and characteristics of change. However, this does not imply that the PERMA elements operate independently or correspond mechanically. Rather, we used them as a flexible interpretive framework to understand the complex and interactive nature of well-being experiences.

These results suggest that the classical reading experience functions as an educational process that promotes well-being at emotional, relational, cognitive, and ontological levels, going beyond simple learning activities. This finding suggests that classical reading enhances cognitive skills and subject understanding while supporting learners’ inner transformation and personal growth. From this perspective, educators must shift their teaching and learning philosophy to focus on how individual learners experience classical reading and how it stimulates their well-being rather than treating classics as mere educational content.

Although the three perception types identified in this study all showed positive responses to classical reading, their approaches and underlying mechanisms differed across cognitive expansion, emotional empathy, and ontological exploration. These differences serve as key variables that influence the effectiveness of classical reading activities. Therefore, instructional design should consider these distinctions and provide diverse participatory structures, expressive methods, and interpretive spaces aligned with learners’ ways of engagement. For example, analytical schematization or concept-centered organization may be meaningful for type 1, while emotional sharing and interaction-based activities with peers are effective for type 2. For type 3, reflective writing and symbolic reconstruction that connect self-narratives with the meaning of life can deepen the classical reading experience.

For classical reading to function as a process that fosters well-being through the interconnected experiences of emotional, cognitive, and existential growth, the role of the learning environment and facilitator is essential. Instructors should guide students to discover life-related directions within classical texts, not as central interpreters but as facilitators who support the construction of meaning. Through such processes, classical reading can function as a tool for knowledge acquisition and reading training, as well as a practical approach to enhancing students’ well-being. The role of classical reading in promoting well-being highlights the need for universities to support psychological well-being and personal growth alongside academic achievement, moving beyond the traditional boundaries of educational objectives.

This study empirically demonstrates that classical reading can contribute to the well-being of STEM students, clarifying its educational significance and practical applicability. Future research should explore the unique features of each identified perception type and expand the investigation of classical reading experiences across different academic disciplines, generational cohorts, and educational settings. Comparative analyses between classical texts and other genres, such as contemporary fiction, nonfiction, or popular literature, may further clarify the distinctive influence of classical works on student well-being. Researchers could also extend these comparisons to earlier educational stages, such as secondary education, to achieve a deeper understanding of how the effects of classical reading may differ by developmental level.

The perception types identified in this study reflect the specific educational context of STEM students, but similar experiential structures may exist in other academic or cultural groups. Cross-disciplinary and cross-cultural studies, including those involving humanities and social science students or international participants, could illuminate the generalizability and contextual specificity of classical reading experiences.

Additionally, exploring the relationship between emotional well-being and academic achievement could offer a more comprehensive understanding of the educational effects of literary engagement.

Future research may also benefit from incorporating quantitative psychological indicators or mixed-methods approaches to assess the impact of classical reading on well-being.

Finally, the implications of this study extend beyond educational settings. The processes of self-reflection, emotional regulation, and value exploration encouraged by classical reading are applicable in various professional and organizational contexts. Literature-based programs could support leadership development, emotional resilience training, and the creation of inclusive organizational cultures. These broader applications warrant further investigation.

## 5. Limitations

This study, consistent with the characteristics of Q methodology, does not aim for generalizability. Using purposive sampling, we strategically selected a small number of participants whose experiences aligned with the research purpose. As such, the findings may not be directly applicable to the broader college student population or students in other academic disciplines.

The analysis focused on participants’ subjective perceptions of classical reading, without incorporating objective indicators of well-being or academic performance. Since Q methodology does not capture broader contextual experiences, future studies could complement this approach with narrative inquiry, case studies, or ethnographic methods to provide a more comprehensive understanding of the topic. While the PERMA model provides a useful interpretive framework, we did not use it as a direct measurement tool. Therefore, we caution researchers about drawing generalized conclusions about well-being outcomes.

This study included STEM students only at a single university, and the number of participants varied across perception types. Although this sampling strategy ensured contextual consistency and comparability, it restricts the generalizability of the findings across institutions. Moreover, the study used a cross-sectional design, which makes it challenging to assess changes over time. Longitudinal research, including follow-up interviews or repeated Q-sorting, would be valuable for exploring the sustainability and developmental trajectory of well-being-related changes.

## 6. Conclusions

This study used the Q methodology to examine how STEM college students perceive the classical reading experience and to identify the characteristics of the perception types associated with this experience. The findings revealed that students recognized classical reading as a multidimensional experience, involving cognitive expansion, emotional engagement, and philosophical reflection. For STEM students, who often view reading as peripheral to their academic training, classical texts offered a unique space for self-reflection and internal growth.

The analysis identified three distinct types of perception. Type 1, the exploratory type, engages in classical reading to broaden thinking and foster analytical insight, often linking the experience to self-development and a sense of accomplishment. Type 2, the experience-oriented type, emphasizes shared enjoyment and emotional resonance through collaborative reading. Type 3, the insight type, views classical reading as a path to exploring universal values and existential questions.

These findings empirically demonstrate that classical reading contributes to diverse pathways of well-being, depending on individual interpretive orientations. It supports emotional resilience, relational connection, and a deepened sense of meaning. This study further highlights the pedagogical significance of classical reading in STEM contexts, showing that it fosters creative thinking and personal growth within learning environments typically dominated by technical rationality. Based on these insights, we recommend designing reading education in STEM curricula to enhance academic and psychological well-being.

## Figures and Tables

**Table 1:** Q methodology research procedures.

Research Step	Period	Details
Q Concourse	June 2023–November 2023	- Collected classical reading-related expressions from academic sources, student reflection papers, and course materials. - Extracted 175 preliminary statements from student assignments, presentation materials, and test responses. - Organized based on the five elements of the PERMA model. - Categorized into 10 subdomains.
Q Sample	December 2023–August 2024	- Eliminated duplicates and ambiguous/overlapping expressions. - Reduced to 55 items after expert review by two Q-methodology specialists. - Final selection of 31 representative statements after expert review by four specialists in Q methodology, focusing on topic balance and expression clarity.
P Sample	October–November 2024	- Participants recruited through bulletin boards in STEM colleges after IRB approval. - Recruited from STEM students who had previously taken a classical reading course. - Final selection of 39 students enrolled in classical reading courses. - Considered participant diversity in gender and academic year.
Q-Sorting	November 2024	- Participants conducted Q-sorting using a forced distribution method (−4 to +4). - Used an 11-point distribution scale, from “most disagree” to “most agree”. - Participants provided written explanations for extreme scores (±4).
Analysis	December 2024–January 2025	- Data analyzed using KenQ Analysis 2.0.1. - Principal component analysis with varimax rotation applied. - Extracted three types based on Z-scores and factor weights. - Comparative analysis conducted with open-ended comments from high-weight participants.

**Table 2:** Eigenvalues and explained variances for the three types.

Type	Type 1	Type 2	Type 3
Eigenvalues	13.52561	3.449888	2.255995
% Explained Variance	35	9	6
Cumulative % Explained Variance	35	44	50

**Table 3:** Demographic background and factor weights by type.

Type	No.	Weight	Gender	Age	Major (Department)	Academic Year
Type 1	P9	10	F	22	Advanced Materials Engineering	3
	P24	6.22810	F	21	Mechanical Engineering	2
	P5	5.16591	F	25	Applied Chemistry	3
	P39	4.64351	F	22	Biomedical Engineering	3
	P27	4.38846	F	24	Smart Farming Science	3
	P3	4.14189	M	19	Industrial & Management Engineering	1
	P29	3.78724	M	25	Computer Engineering	4
	P22	3.32210	M	23	Mechanical Engineering	2
	P37	3.10138	M	23	Electronic Engineering	2
	P12	3.09931	M	24	Mechanical Engineering	3
	P23	3.02008	M	25	Applied Chemistry	4
	P25	3.00693	M	26	Electronic Engineering	3
	P16	2.92837	M	23	Chemical Engineering	4
	P7	2.89914	F	22	Applied Chemistry	4
	P15	2.81797	M	23	Mechanical Engineering	3
	P20	2.68503	F	23	Oriental Medicine Biotechnology	3
	P28	2.60919	M	21	Architectural Engineering	2
	P10	2.23275	M	23	Architecture	4
	P1	2.15059	F	20	Nuclear Engineering	1
Type 2	P38	7.67231	M	28	Civil Engineering	2
	P19	6.30840	M	25	Electronic Engineering	4
	P4	5.54050	F	19	Genetics & Biotechnology	1
	P21	4.71814	F	23	Oriental Medicine Biotechnology	4
	P32	4.40140	M	24	Computer Engineering	3
	P13	4.38358	M	22	Electronic Engineering	2
	P34	4.02570	M	26	Astronomy & Space Science	4
	P17	3.49965	F	22	Electronic Engineering	3
	P30	2.49445	M	20	Chemical Engineering	1
	P11	2.29903	M	24	School of Computing	3
Type 3	P31	6.12539	F	23	Astronomy & Space Science	3
	P26	5.13532	M	23	Food Science & Biotechnology	2
	P14	4.34327	M	25	Food Science & Biotechnology	4
	P36	4.05035	F	22	Chemical Engineering	3
	P33	3.80328	M	25	Electronic Engineering	3
	P6	3.67105	M	28	Electronic Engineering	4
	P8	3.57045	F	24	Applied Chemistry	4
	P35	2.76769	M	23	Environmental Science & Engineering	4
	P18	2.25843	M	26	Advanced Materials Engineering	4
	P2	1.10316	M	25	Industrial & Management Engineering	4

**Table 4:** Exploratory type that broadens thinking through diverse perspectives (type 1).

No.	Statement	Z-Score	Q-Sort Value	Distinguishing (*p* < 0.01)
2	Classical reading leads to an exploration of human nature.	1.99	+4	
3	Classical reading rejects the absoluteness of right and wrong.	0.97	+2	*
4	Reading classics together is more rewarding than reading alone.	0.85	+2	*
5	Classical reading provides rest and relaxation.	−0.83	−2	*
8	Classical reading helps me understand others.	1.53	+3	*
9	Classical reading cultivates an attitude of living together.	1.17	+3	*
11	Classical reading helps me set the direction of my life.	0.02	+1	*
12	Classical reading reveals hidden aspects of myself.	−0.08	0	*
14	Classical reading encourages multiple perspectives on events or situations.	1.97	+4	
16	Classical reading helps me realize I am the main character in my life.	−0.64	−2	*
18	Classical reading is an enjoyable activity.	−0.12	0	*
19	Classical reading strengthens my sense of challenge	−1.15	−3	
21	Classical reading broadens my perspective on the world.	1.42	3	
22	Classical reading is the greatest gift I can give myself.	−1.13	−3	*
26	Classical reading helps relieve the stress of college life.	−1.04	−3	*
27	Classical reading provides practical knowledge.	−1.54	−4	*
28	Classical reading improves my decision-making skills.	−0.19	0	*
29	Classical reading interferes with my major studies.	−2.31	−4	
31	Classical reading is the best way for me to achieve personal growth.	−0.48	−1	*

Note. The table includes statements with Z-scores of ≥+1.00 and ≤–1.00; * indicates *p* < 0.01; the table also includes distinguishing statements even if Z-scores do not meet the ±1.00 threshold.

**Table 5:** Experience type that shares achievement and enjoyment through reading together (type 2).

No.	Statement	Z-Score	Q-Sort Value	Distinguishing (*p* < 0.01)
2	Classical reading leads to an exploration of human nature.	0.89	2	*
4	Reading classics together is more rewarding than reading alone.	2.1	+4	*
5	Classical reading provides rest and relaxation.	1.2	+3	*
9	Classical reading cultivates an attitude of living together.	0.47	+1	*
10	Classical reading supports communication across generations.	0.39	+1	*
13	Classical reading makes me reflect on my past.	−1.29	−3	*
14	Classical reading encourages multiple perspectives on events or situations.	1.38	+3	
16	Classical reading helps me realize I am the main character in my life.	−1.36	−4	
19	Classical reading strengthens my sense of challenge.	−0.23	0	*
21	Classical reading broadens my perspective on the world.	1.06	3	
22	Classical reading is the greatest gift I can give myself.	0.07	+1	*
23	Classical reading breaks down my fixed ideas.	−0.33	−1	*
29	Classical reading interferes with my major studies.	−2.23	−4	
30	Classical reading allows me to feel a sense of accomplishment.	1.91	+4	*

Note. The table presents statements with Z-scores of ≥+1.00 and ≤–1.00; * indicates *p* < 0.01; the table includes distinguishing statements even if Z-scores do not meet the ±1.00 threshold.

**Table 6:** Insight type that seeks universal values and truth (type 3).

No.	Statement	Z-Score	Q-Sort Value	Distinguishing (*p* < 0.01)
2	Classical reading leads to an exploration of human nature.	1.85	+4	
3	Classical reading rejects the absoluteness of right and wrong.	−1.16	−3	
4	Reading classics together is more rewarding than reading alone.	0.34	+1	*
5	Classical reading provides rest and relaxation.	0.39	+1	*
6	Classical reading is especially necessary for STEM students.	−1.14	−3	*
7	Classical reading teaches how to express emotions.	−0.95	−2	*
9	Classical reading cultivates an attitude of living together.	−0.37	−1	*
11	Classical reading helps me set the direction of my life.	1.35	+3	*
12	Classical reading reveals hidden aspects of myself.	−1.05	−2	
14	Classical reading encourages multiple perspectives on events or situations.	1.71	+3	
15	Classical reading supports the formation of identity.	0.62	+2	*
16	Classical reading helps me realize I am the main character in my life.	−1.65	−4	
19	Classical reading strengthens my sense of challenge.	−1.06	−3	
20	Classical reading gives me the courage to solve my problems.	−1	−2	
21	Classical reading broadens my perspective on the world.	2.08	+4	*
22	Classical reading is the greatest gift I can give myself.	−0.55	−1	*
29	Classical reading interferes with my major studies.	−1.51	−4	*

Note. The table presents statements with Z-scores of ≥+1.00 and ≤–1.00; * indicates *p* < 0.01; the table includes distinguishing statements even if Z-scores do not meet the ±1.00 threshold.

## Data Availability

The data are available from the corresponding author upon reasonable request.

## Appendix A

**Table A1:** Q-Statements with Z-Scores and Q-Sort Values by Type.

No.	Statements	Factor 1		Factor 2		Factor 3	
		Z-Score	Q-Sort Value	Z-Score	Q-Sort Value	Z-Score	Q-Sort Value
1	Classical reading closely relates to modern life.	0.8	2	0.06	0	0.61	2
2	Classical reading leads to an exploration of human nature.	1.99	4	0.89	2 *	1.85	4
3	Classical reading rejects the absoluteness of right and wrong.	0.97	2 *	−0.62	−1	−1.16	−3
4	Reading classics together is more rewarding than reading alone.	0.85	2 *	2.1	4 *	0.34	1 *
5	Classical reading provides rest and relaxation.	−0.83	−2 *	1.2	3 *	0.39	1 *
6	Classical reading is especially necessary for STEM students.	0.31	1	0.79	2	−1.14	−3 *
7	Classical reading teaches how to express emotions.	−0.28	−1	−0.23	0	−0.95	−2 *
8	Classical reading helps me understand others.	1.53	3 *	0.9	2	0.87	3
9	Classical reading cultivates an attitude of living together.	1.17	3 *	0.47	1 *	−0.37	−1 *
10	Classical reading supports communication across generations.	0.25	1	0.39	1 *	0.55	1
11	Classical reading helps me set the direction of my life.	0.02	1 *	−0.67	−2	1.35	3 *
12	Classical reading reveals hidden aspects of myself.	−0.08	0 *	−0.91	−3	−1.05	−2
13	Classical reading makes me reflect on my past.	0.3	1	−1.29	−3 *	0.31	0
14	Classical reading encourages multiple perspectives on events or situations.	1.97	4	1.38	3	1.71	3
15	Classical reading supports the formation of identity.	−0.21	0	−0.64	−1	0.62	2 *
16	Classical reading helps me realize I am the main character in my life.	−0.64	−2 *	−1.36	−4	−1.65	−4
17	Classical reading shifts my focus from comparing with others to focusing on myself.	−0.5	−2	−0.9	−2	−0.56	−1
18	Classical reading is an enjoyable activity.	−0.12	0 *	0.96	2	0.63	2
19	Classical reading strengthens my sense of challenge.	−1.15	−3	−0.23	0 *	−1.06	−3
20	Classical reading gives me the courage to solve my problems.	−0.45	−1	−0.64	−2	−1	−2
21	Classical reading broadens my perspective on the world.	1.42	3	1.06	3	2.08	4 *
22	Classical reading is the greatest gift I can give myself.	−1.13	−3 *	0.07	1 *	−0.55	−1 *
23	Classical reading breaks down my fixed ideas.	0.36	2	−0.33	−1 *	0.32	1
24	Classical reading has enhanced my imagination.	−0.56	−2	−0.17	0	0.17	0
25	Classical reading gives me the strength to overcome pain.	−0.24	−1	−0.6	−1	0.04	0
26	Classical reading helps relieve the stress of college life.	−1.04	−3 *	0.01	0	−0.02	0
27	Classical reading provides practical knowledge.	−1.54	−4 *	−0.91	−3	−0.99	−2
28	Classical reading improves my decision-making skills.	−0.19	0 *	−0.8	−2	−0.81	−1
29	Classical reading interferes with my major studies.	−2.31	−4	−2.23	−4	−1.51	−4 *
30	Classical reading allows me to feel a sense of accomplishment.	−0.18	0	1.91	4 *	0.23	0
31	Classical reading is the best way for me to achieve personal growth.	−0.48	−1 *	0.32	1	0.76	2

Note. The authors originally developed all Q-statements in Korean and translated them into English, with a professional editor reviewing the translations to preserve the original meaning. An asterisk (*) indicates distinguishing statements that are statistically significant at *p* < 0.01, reflecting items uniquely associated with a specific factor.
